# Nature in the Cure of Disease

**Published:** 1862-01

**Authors:** 


### NATURE IN THE CURE OF DISEASE.

Reprinted from the *Boston Med. and Surgical Journal.*

The fashion, that is somewhat prevalent at the present time, of decrying the use of drugs in the treatment of disease, and which arises partly from a skepticism characteristic of our days, and probably in some degree from a species of indolence which readily finds an excuse for getting rid of a study, to many an unwelcome task, is well commented upon in the following extract from an article in a recent number of the London *Medical Times and Gazette*, which we would commend to our readers as containing much sound sense:—

"Another insidious and plausible mode of discountenancing the use of drugs is to represent them as 'unnatural,' and to speak of 'Nature and Art in the cure of disease,' as if there were some antagonism between them, and as if the use of drugs were artificial, and, if so, reprehensible.

"Just as one portion of popular error arises from ignorance of facts, so does another and more inveterate set of errors arise from confusion in the use of words. 'Nature,' for example, is a word that is incessantly quoted. It is 'natural,' we are told, to wear the beard; 'natural' to drink when thirsty; 'natural' for mothers to suckle their infants; and, as the authors of twopenny treatises, and lecturers on diet never fail to tell us, it is 'natural' to eat brown bread. Popular books on medicine are rich in this sort of practical joke, if we may call anything a joke that destroys human life, for we hold that bad logic destroys more lives than gunpowder. For example: one of the popular books on medicine which we reviewed lately (and by no means the worst of them) contained a story such as this:—'A monthly nurse once asked me, if she should give some gruel to a newly-born infant. I replied, "Now don't you think, nurse, that if *Nature had intended* it to have gruel, the child would have been born with a bottle of gruel round its neck?"' This poor woman was vanquished by this precious piece of argument; yet she might fairly have asked, in return, had Nature intended the cord to be divided; had she meant the child to be washed and dressed, and whether scissors, thread, hot water, soap, sponge, violet-powder, and cambric chemisettes might not have been objected to on the ground of unnaturalness, quite as much as gruel. The fact is, that the word *Nature* is used to signify at least two independent ideas. One is that brute, naked state in which any given thing happens to be found without interference or improvement by the hand of man: *state of Nature* as it is called. The other meaning includes the whole faculties and capabilities, including the circumstances favorable to full and luxuriant growth and development. Thus, man in a state of Nature (to use the word in one sense) is a filthy, stinking, verminous savage, thoroughly selfish and utterly deficient in those finer feelings of love for parents and children which we, educated under the influence of Christianity, are wont to call 'natural affections.' But the nature of man (to use the word in the other sense), includes the possession of conscience and reason, which teach him to check mere brutal instincts, and prompt him to explore, subdue and utilize all the objects he meets with, and to employ them in such a way as to produce for himself the greatest amount of beauty, comfort, health and strength. Hence to denounce or sneer at, as *unnatural*, the use of drugs, which man's instincts prompt him to seek, and his intellect enables him to find, is a monstrous and most mischievous perversity. But so it is. A patient in a well-built house, in a comfortable bed, fed with food and clothed with textures from all the quarters of the globe, and dependent for his comforts, and even his life, on the accumulated products of centuries of human art, is supposed to be treated *naturally* if he takes no medicine: the case is 'left to Nature;' but if any of those beneficent means be used which have also been slowly gathered together during the progress of human civilization—leeches, for example, to take a little blood where it is superfluous; anodynes to procure sleep, or aperients to empty the bowels—forsooth this is unnatural and artificial! and therefore suspicious, if not positively wrong.

"Let us say, emphatically, to administer drugs out of mere routine is contemptible. To give unnecessary medicines for the sake of adding to professional profit is degrading. The rash and blindfold heroic practice of giving active remedies in all cases (whether bleeding or brandy), is dangerous. To neglect air, food and regimen, is to let half our weapons lie idle. But we do desire most earnestly to vindicate and uphold the rational and temperate use of those drugs which are employed in ordinary practice; because they can produce effects quickly, which cannot be obtained quickly from rest, diet, or other appliances, and because human suffering may be enormously mitigated by them. Whilst we get rid of the old apothecary traditions, let us avoid that half-indolent, half-skeptical spirit that would rob us of some of our most valuable instruments, and encourage the public in prejudices that have been but too successfully instilled by our adversaries. Let us study the practical art of healing, and the uses of drugs especially, for, in the words of the author of Ecclesiasticus, 'He that is wise will not despise them.'"

